# RTJTN: Relational Triplet Joint Tagging Network for Joint Entity and Relation Extraction

**DOI:** 10.1155/2021/3447473

**Published:** 2021-10-16

**Authors:** Zhenyu Yang, Lei Wang, Bo Ma, Yating Yang, Rui Dong, Zhen Wang

**Affiliations:** ^1^The Xinjiang Technical Institute of Physical and Chemistry, Chinese Academy of Sciences, Urumqi 830011, China; ^2^University of Chinese Academy of Sciences, Beijing 100049, China; ^3^Xinjiang Laboratory of Minority Speech and Language Information Processing, Urumqi 830011, China

## Abstract

Extracting entities and relations from unstructured sentences is one of the most concerned tasks in the field of natural language processing. However, most existing works process entity and relation information in a certain order and suffer from the error iteration. In this paper, we introduce a relational triplet joint tagging network (RTJTN), which is divided into joint entities and relations tagging layer and relational triplet judgment layer. In the joint tagging layer, instead of extracting entity and relation separately, we propose a tagging method that allows the model to simultaneously extract entities and relations in unstructured sentences to prevent the error iteration; and, in order to solve the relation overlapping problem, we propose a relational triplet judgment network to judge the correct triples among the group of triples with the same relation in a sentence. In the experiment, we evaluate our network on the English public dataset NYT and the Chinese public datasets DuIE 2.0 and CMED. The F1 score of our model is improved by 1.1, 6.0, and 5.1 compared to the best baseline model on NYT, DuIE 2.0, and CMED datasets, respectively. In-depth analysis of the model's performance on overlapping problems and sentence complexity problems shows that our model has different gains in all cases.

## 1. Introduction

Joint extraction of entity and relation is an indispensable work for processing unstructured text information and constructing knowledge graphs, which aims to extract all relational triplets in the text. The form of relational triplets is (*subject*, *relation*, *object*), for example (Washington, Capital of, America).

**Figure 1:**
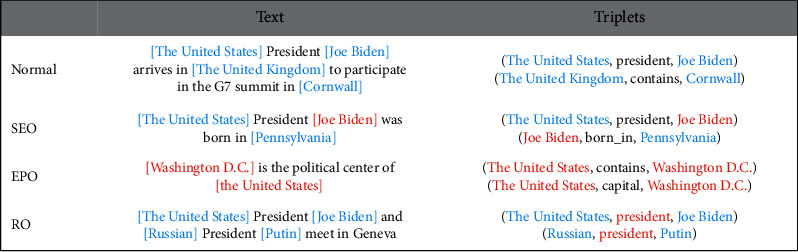
Examples of the normal, EntityPairOverlap (EPO), SingleEntiyOverlap (SEO), and RelationOverlap (RO). The overlapping entities and relations are masked in red.

Early work used the pipeline extraction method [1, 2] to extract entities and relations separately. But the pipeline method ignores the connection between entity extraction and relation extraction. Therefore, a lot of work in recent years has focused on the joint extraction of entities and relations, like methods based on artificially constructed features [3–5] and neural network methods [6–8]. With the development of deep learning and the application of pretrained models, entity and relation extraction has reached a new level of performance [9, 10]. But joint extraction makes the task complicated and many new problems appear, like EntityPairOverlap (EPO), SingleEntiyOverlap (SEO), and RelationOverlap (RO) (see Figure 1).

In order to solve the entities and relations overlapping problems, many researchers have proposed solutions. Therefore, many excellent models have emerged to solve the overlapping problems. For example, Zeng et al. [11] proposed a joint entities and relations extraction model based on the seq2seq method; Nayak and Ng [12] applied the encoder-decoder to the entities and relations joint extraction framework; and Wei et al. [8] proposed that relation is a learnable formula from subject to object. These models have excellent results in extraction of entities and relations, but they have a common shortcoming: artificially decomposing extraction of entities and relations into multiple subtasks. They still decompose the extraction of entities and relations into several subtasks in the end-to-end model. The problem with this approach is that the entity extraction task and the relation extraction task are only exchanging a small amount of information through artificial design and the entity extraction task's error will directly affect the relation extraction.

In this paper, we present a new entities and relations tagging method that can tag all entities and relations information in one label. Our tagging method can turn the extraction of entities and relations model into a simple sequence tagging model and all entities and relations information can be output at one time. Given a sentence, we tag each word with a new label that is divided into 5 regions: the head of the subject (S-H), the tail of the subject (S-T), the head of the object (O-H), the tail of the object (O-T), and the nonentity word region (O). For each region in the label, we are inspired by the binary tagging model to add predefined relations information to each region outside the nonentity word region and use binary 0,1 to represent the discrimination result. This tagging method is a good solution to EPO and SEO problems.

In addition to EPO and SEO problems, our paper formally discusses the RO problem (see Figure 1) for the first time. In the RO problem, multiple relational triplets are sharing the same relation. However, multiple triplets of the same relation have similar contexts, which results in the entity embeddings in the triplets becoming also very similar. Therefore, in the RO problem, models are difficult to identify the subject and object in the same triplet. The RO problem has been ignored by previous work. Most works only use heuristic methods to combine the closest subject and object [11, 13]. For solving RO problem, we propose a relational triplet judgment network to distinguish the subject and object of the same relation.

In summary, this work's main contributions are as follows:

1. We present a joint tagging method for entities and relations in sentence. This method not only enables the complete integration of entity information and relational information but also addresses EPO and SPO problems.
2. We evaluate the model using the relation overlapping problem, which is where multiple triplets in a sentence share the same relation for the first time; and we present a relational triplet judgment network for the RO problem. Our model has achieved good results on the dataset for this problem.
3. In the experiment, we evaluate our model on the English dataset NYT [14] and the Chinese datasets DuIE 2.0 and CMED. Our model outperforms the previous models on the three datasets.

## 2. Related Work

The extraction of relational triplets has always been an important task in the field of natural language processing. It can not only extract knowledge from the unstructured text but also provide prior knowledge for many important tasks of artificial intelligence, like Question Answering System, Knowledge Graph, and Machine Translation.

In early work, the problem of relational triplet extraction is mainly solved by pipeline method, which first identifies all entities in the text and then identifies all relations between any two entities. Nadeau and Sekine [1] and Zelenko et al. [2] used a pipeline to identify entities and relations in the text for the first time; but pipeline method ignores the connection between the two tasks and does not play a role in mutual optimization. To solve this problem, many papers have proposed joint extraction models of entities and relations. Ren et al. [5], Li and Ji [4], and Miwa and Sasaki [15] proposed joint extraction models of entity and relations based on artificially constructed features; but artificial feature construction is difficult and model performance is unstable. Zheng et al. [16] used the LSTM network to realize the joint extraction of entities and relations and used the heuristic principle of proximity to combine the subject and the object to solve RelationOverlap (RO) problem. Zeng et al. [11] proposed that the extraction model of entities and relations needs to solve the overlapping problems, EntityPairOverlap (EPO) and SingleEntiyOverlap (SEO), at the first time.

In order to solve the entities and relations overlapping problems, many papers have proposed solutions. Fu and Ng [17] added the graph convolutional network to the entities and relations joint extraction model. Nayak and Ng [12] added the idea of encoder-decoder to the model of joint extraction of entities and relations. Bekoulis et al. [18] transformed the task of extracting entities and relations into a multihead selection task. Yu et al. [19] added a span-based tagging strategy and layered decoding strategy to the joint extraction task. Liu et al. [20] extracted entities based on conditional random fields and judged relation based on supervised multihead self-attention. Wei et al. [8] proposed that relations are constructed as a function from subject to object. Although these methods use end-to-end method to integrate entity extraction and relation extraction into a whole, the entity and relation are divided into several subtasks within the model. Our joint entities and relations tagging method can completely merge entity extraction and relation extraction into one task.

## 3. Relational Triplet Joint Tagging Network

In this section, we will introduce our relational triplet joint tagging network. First, we will elaborate on joint entities and relations tagging method, which can convert the joint extraction of entities and relations into a sequence tagging problem and output result at one time. Our tagging method can solve the EPO and SEO problems very well. Then we will introduce the relational triplet judgment network for the RO problem, which can integrate sentence information, entity semantic information, and location information to judge whether the triples in the joint tag are correct.

### 3.1. Joint Entities and Relations Tagging Layer

**Figure 2:**
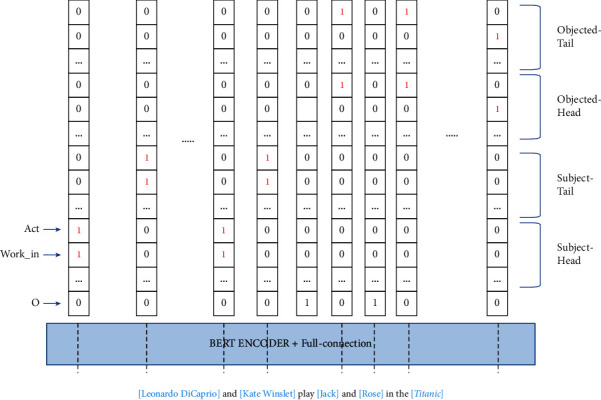
The joint tagging method of entities and relations. The label of each word is divided into five regions, and the other four regions except the O region are divided into the number of relation label.

In order to eliminate the error between entity extraction and relation extraction, we design a joint tagging method of entities and relations to enable the model to output all information at one time instead of processing it separately. We treat the task of entities and relations joint extraction as a sequence tagging task. For a sentence, each word corresponds to a label with entities information and relations information (see Figure 2). For entities information, we divide the label of each word into five regions: the head of the subject (S-H), the tail of the subject (S-T), the head of the object (O-H), the tail of the object (O-T), and the nonentity words (O). For the entity in each triplet, we only tag the head and tail of the entity and tag the other parts of the entity with O. For relation information, we divide the other four regions except the O region into labels for the number of relations. In other words, the length of the label corresponding to each word is 4*N*+1, where *N* is the number of preset relations. We are also inspired by the binary tagging method [8]. When the network recognizes the subject of a relation, we tag the position of the corresponding relations in the S-H and S-T region as 1, and when the object is recognized, we make the same label in the O-H and O-T regions. Since each entity can be in multiple relational triplets or both subject and object, there may be multiple labels with a value of 1 of each word, like Leonardo is in both (Leonardo DiCaprio, Act, Jack) and (Leonardo DiCaprio, Work_in, Titanic) (see Figure 2). In other words, the task of tagging each word is a multiclassification task. This tagging approach can be a good solution to EPO and SEO problems.

### 3.2. Relational Triplet Judgment Network

**Figure 3:**
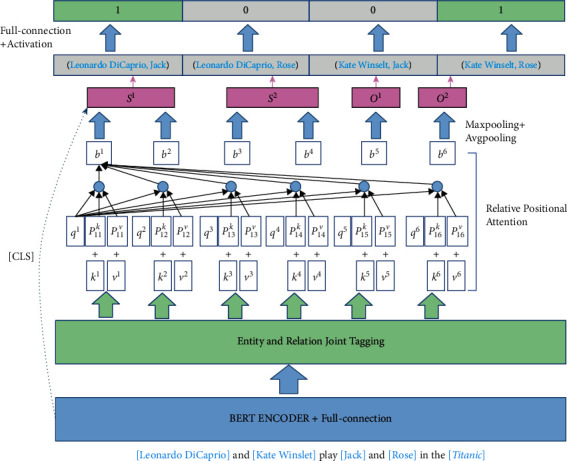
Overview of triplet judgment network with entity and relation joint tag. In this example, two relational triplets have a relation overlapping (RO) problem.

In order to solve the RO problem, we propose a relational triplet judgment network (see Figure 3). First, we extract the subject and object words encoded by the pretrained model based on the results of the joint entity and relationship labels. Then, we use relative position concerns to embed the words of the subject and object into the relative position information. We combine the subject and the object in pairs and add the sentence information to determine whether the subject and object are a triplet.

#### 3.2.1. BERT Encoder

The encoder can extract features from the sentence information *S*_*i*_ and convert *S*_*i*_ into a word embedding *X*_*i*_. The output word embedding *X*_*i*_ can be used for the prediction and tagging of subsequent modules. We use BERT [21, 22] encoder to extract features from sentence information.

Here we briefly review the overview of BERT. BERT is a language representation model composed of a multilayer bidirectional transformer [23] encoder. Through self-supervised training on a large number of unmarked corpora, the BERT model contains rich language knowledge. In the training process, BERT learns the deep representation of words by randomly masking or replacing some words and predicting through context and has achieved surprising results in multiple tasks. We denote the BERT model as *B*(*x*) and denote each layer of the BERT model as *T*(*x*). The operation process of the BERT can be expressed as

(1) $$\begin{array}{c} H=B\left(E_{t}+E_{p}+E_{s}\right), \end{array}$$

and the operation of each layer of BERT can be expressed as

(2) $$\begin{array}{c} H^{0}=T\left(WO_{t}+E_{p}+E_{s}\right),\\ H^{l}=T\left(H^{l-1}\right), \end{array}$$

where *E*_*t*_ is word split embedding, *E*_*p*_ is position embedding, *E*_*s*_ is sentence segmentation embedding, *O*_*t*_  is one-hot embedding of input words, and *W* is the one-hot embedding stored by BERT.

#### 3.2.2. Relative Positional Attention

The relative position is important information for judging whether the subject and the object are in the same triplet. Generally, the length of the relative position distance between entities in the same relational triplet is shorter than the distance of the relative position with other entities. But not all subjects and objects in the same triplet are close. In order to solve this problem, inspired by relative position representations in machine translation [24], we add an attention layer with learnable relative positional embedding in the output layer of the encoder.

On the basis of the attention mechanism, we add the relative position information between the subject and the object. Specifically, in order to obtain more comprehensive position information, we use two vectors to represent the relative positional encoding between each word and learn the relative positional information of the entity character level while calculating the subject and object attention. We use the vectors *P*_*so*_^*k*^ and *P*_*so*_^*v*^ to represent the relative positional information between the subject S and the object O. The relative positional vectors *P*_*so*_^*k*^ and *P*_*so*_^*v*^ add to the calculation process of the key and value in the attention, respectively. The detailed operation is as follows:

(3) $$\begin{array}{c} \alpha_{so}=a_{s}W^{q}\cdot \left(a_{s}W^{k}+P_{so}^{k}\right),\\ \alpha_{so}^{\prime }=\text{Softmax}\left(\alpha_{so}\right),\\ b_{s}=\sum_{o=1}^{n}\alpha_{so}^{\prime }\left(a_{o}W^{v}+P_{so}^{v}\right), \end{array}$$

where *W*^*q*^, *W*^*k*^, and *W*^*v*^ represent the weight matrix of query, key, and value in the attention mechanism, respectively. *a*_*s*_ and *a*_*s*_ represent the word embedding in the subject entity and the object entity, respectively. We set a limit for the relative position; that is, when the relative distance of the word exceeds the set maximum length, we treat it as the maximum distance. The maximum distance we set is 50. The detailed operation is as follows:

(4) $$\begin{array}{c} P_{so}^{k}=W_{\text{len}\left(o-s,L_{\max}\right)}^{k},\\ P_{so}^{v}=W_{\text{len}\left(o-s,L_{\max}\right)}^{v},\\ \text{len}\left(x,L_{\max}\right)=\max\left(L_{\max}\;,\text{ abs}\left(x\right)\right), \end{array}$$

where *L*_max_ is our maximum distance.

#### 3.2.3. Entity Feature Extraction

Through the joint tagging of entities and relations and relative positional attention, we can get all the subjects and objects in the sentence with relative position information and relations information. In order to extract the feature of the entities as comprehensively as possible, we extract each subject and object through two processes of average pooling and maximum pooling; and, for the entity embedding to fuse the global feature of the sentence instead of the feature of the surrounding words, we concatenate the [CLS] from the BERT as sentence embedding into the entity embedding. Then, we enumerate all possible combinations of subject and object in the same relation and judge whether it is the correct combination. The specific operation is as follows:

(5) $$\begin{array}{c} S=\text{Concat}\left(\text{MaxPooling}\left(\sum_{i=1}^{s}S_{i}\right),\text{AvgPooling}\left(\sum_{i=1}^{s}S_{i}\right),E_{cls}\right),\\ O=\text{Concat}\left(\text{MaxPooling}\left(\sum_{j=1}^{o}O_{j}\right),\text{AvgPooling}\left(\sum_{j=1}^{o}O_{j}\right),E_{cls}\right),\\ E_{so}=\text{Concat}\left(S,O\right),\\ E_{so}^{\prime }=\text{Sigmoid}\left(WE_{so}+b\right), \end{array}$$

where *S* and *O* are subject and object, respectively. *S*_*i*_ and *O*_*j*_ are word embedding in a subject and an object, respectively. *E*_cls_ is the output vector at [CLS] in BERT.

### 3.3. Loss Function

Our model is divided into two stages. In the first stage, we use BECWithLogits Loss to learn the joint tagging of entities and relations. In order to reduce the influence of sparse tags on model learning, we square the probability value output by the model to make the result smoother. In the second stage, the relational triplet judgment network uses CrossEntropy Loss. The losses of these two stages are added together in a certain proportion and jointly trained.

(6) $$\begin{array}{c} {\text{Loss}}_{\text{CE}}=-\left[y_{n}*\;\log\left(x_{n}\right)+\left(1-y_{n}\right)*\;\log\left(1-x_{n}\right)\right],\\ \alpha =\left\{\begin{array}{cc} 1, & x_{n}<0,\\ 2, & x_{n}\geq 0, \end{array}\right.\\ x_{n\_\text{smooth}}={\left(-1\right)}^{\alpha }*{\left(x_{n}\right)}^{2},\\ {\text{Loss}}_{\text{BCE}}=-\left[y_{n}*\;\log\left(\sigma \left(x_{n\_\text{smooth}}\right)\right)+\left(1-y_{n}\right)*\;\log\left(1-\sigma \left(x_{n\_\text{smooth}}\right)\right)\right],\\ \text{Loss}=\;{\text{Loss}}_{\text{BCE}}+\lambda *{\text{Loss}}_{\text{CE}}, \end{array}$$

where *x*_*n*_ is the output result of the *n*th batch size, *y*_*n*_ is the label of the *n*th batch size, and *λ* is the ratio of the addition of the loss functions; we set it to 0.001.

## 4. Experiments

In this work, we designed three experiments to evaluate our network. First, we used the entire test set to evaluate the performance of the model to reflect the model's ability to deal with common problems. Then, in order to evaluate the performance of the model in processing complex sentences, we tested the model in test sets with overlapping problems and different numbers of triples.

### 4.1. Datasets

For comparison with the previous models in this field, we select one of the most popular datasets: NYT. For proving our model has good performance in multiple languages, we also evaluate our model on Chinese datasets: DuIE 2.0 and CMED. For showing the differences between the three datasets, we analyze and compare them (see Table 1). NYT is a dataset in the English news field and it contains 25 kinds of relations. DuIE 2.0 is a Chinese general field dataset containing 54 kinds of relations. CMED is a Chinese medical field dataset containing 43 kinds of relations. We also analyze the overlap problem and the number of triples in each dataset. These three datasets have different characteristics that can comprehensively evaluate the performance of the model.

### 4.2. Implementation Details

Our model is implemented using PyTorch and the optimizer of the network framework is AdamW [25]. In terms of model parameters, the batch size in all our experiments is 20, the learning rate is reduced from 0 to 0.0003 and then to 0 as the number of training increases, the maximum sequence length is 256, and the word out of range is ignored. In order to obtain more accurate information in the second stage of the model, we train separately for 10 epochs in the first stage and then train jointly. We use Tesla V100 to train our model for up to 50 epochs and evaluate the model on the validation set. We select the best model and output the final result on the test set. When training on the NYT dataset, the pretrained model we use is BERT-base. When training on the DuIE 2.0 and CMED datasets, the pretrained model we use is BERT-wwm-ext.

### 4.3. Baselines and Evaluation Metrics

We select the advanced models in this field in recent years for comparison. (1) NovelTagging [16] proposed the joint tagging method of entities and relations for the first time, but it did not solve the overlapping problems. (2) CopyRE [11] first proposed using the encoder-decoder structure for the extraction of entities and relations tasks. (3) GraphRel [17] used graph convolutional networks to combine the features of all words to optimize the performance of the model. (4) ETL-Span [19] added the strategy with span information to the extraction model. (5) WDec [12] used seq2seq to generate word sequences. (6) CopyMTL [26] used multitask learning framework to combine extraction of entities and relations. (7) RSAN [13] proposed a relation-specific attention network to solve the overlapping problems. (8) CasRel [8] put forward the concept that the relations are a function of subject to object.

When the triplet output by the model is exactly the same as the label, we judge it as the correct result. We use Precision (Prec), Recall (Rec), and F1 score as indicators of the evaluation model.

### 4.4. Experimental Results and Analysis

#### 4.4.1. Main Results

From the results shown in Table 2, we can find that our model on the NYT dataset surpassed all baseline models in the comprehensive index F1 score; and Table 3 shows the evaluation results of our model and the best baseline model in the Chinese datasets DuIE 2.0 and CMED; our model is still the best in F1 score. The test results prove that the comprehensive performance of our model is better than those of all baseline models. Compared with the best baseline model, the F1 value of our model is improved by 1.1 on the NYT dataset, 6.0 on the DuIE 2.0 dataset, and 5.6 on the CMED dataset; and these three datasets of experiments also prove that the performance of our model is not limited to a single language and it gets the best results under multiple language tests.

We analyze the reasons why RTJTN can get better performance and summarized the following points: (1) Although the SOTA model CasRel has achieved exciting results, it is essentially a two-stage model. The error of extracting entities directly affects the result of extracting relations. Our model combines entity extraction and relation extraction into one stage to eliminate error iteration. (2) The CasRel model does not make obvious feature distinctions for the relational triples in the RO problem but only achieves the goal through the self-learning of the model. Compared with our model, in order to distinguish the features between the relational triples, attention information with relative positions is added. (3) The performance improvement of our model on the DuIE 2.0 dataset and the CMED dataset is much higher than that of the NYT dataset. Our analysis found that the number of relations between the DuIE 2.0 dataset and the CMED dataset far exceeds that of NYT and the average sentence length is also much longer than the sentences in the NYT dataset. We believe that the more there is triple information in the sentence, the greater is the error iteration that CasRel suffers. On the contrary, our model does not have this shortcoming.

#### 4.4.2. Ablation Study of RTJTN

In order to evaluate the contribution of each part of the model to the results, we performed an ablation study on the NYT dataset. Starting from the complete model, we remove part of the structure of the model every time and observe the effect of this structure on the result, which is shown in Table 4. (1) Relation Positional Attention can effectively provide relative position information between subject and object. (2) Information of Sentence enables word embedding information to get richer semantic information. (3) We replaced the original Maxpooling and Average Pooling with the word embedding in the entity directly splicing, and the F1 score dropped significantly. (4) We remove the relational triplet judgment network and only keep the F1 score of the joint entities and relations tagging layer, which shows that our network is of great help in solving the RO problem. After joining relational triplet judgment network training, the F1 score of our joint entities and relations tagging also increased from 88.28 to 83.34, indicating that the relational triplet judgment network is not only helpful to the final result but also helpful to the training of the joint tagging layer.

#### 4.4.3. Analysis on Overlapping Cases

**Figure 4:**
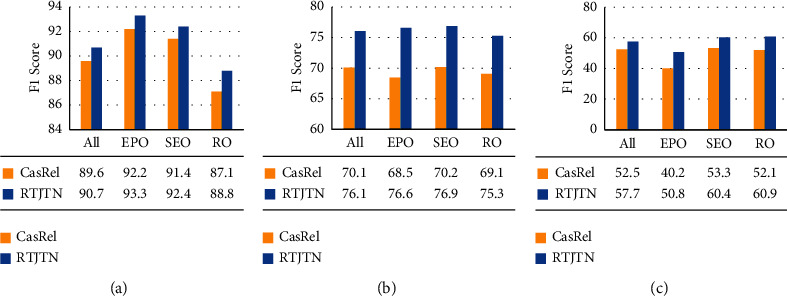
Relation extraction on sentences with overlapping problems. (a) F1 on the NYT dataset. (b) F1 on the DuIE 2.0 dataset. (c) F1 on the CMED dataset.

The overlapping problems of entities and relations impact traditional tagging methods and have a great impact on the final result. In order to prove that our model can effectively solve the overlapping problems, we separately evaluate our model on three datasets of the overlapping problems. We divide the overlapping problems into three types, EntityPairOverlap (EPO), SingleEntiyOverlap (SEO), and RelationOverlap (RO), and extract the data of these three situations from NYT, DuIE 2.0, and CMED datasets. We compare our model with the best baseline model in three cases and the results are shown in Figure 4. From the results in the figure, we can see that our model surpasses the baseline model in all three overlapping problems.

When testing on the RO problem datasets, the performance of the baseline model is worse than the result of testing on the complete data set, which also proves that the RO problem can have a negative impact on the performance of the model. The results in Figure 4 show that when the model predicts sentences with RO problems, the negative impact of our model is significantly less than the best baseline model. This proves that our model can better predict the relational triplets with RO problems.

#### 4.4.4. Analysis on Different Numbers of Relational Triplets

The number of relational triplets in the text also has a huge impact on the results of the extraction task. Generally, the more the number of relational triplets is in a sentence, the more difficult it is to extract the correct relational triplets. In order to evaluate the performance of our model for extracting different numbers of triplets in sentences, we divide the sentences in the NYT, DuIE 2.0, and CMED datasets into five categories, which represent sentences containing 1, 2, 3, 4, and ≥5 triplets in sentences. The results are shown in Table 5. From the results in the table, we can find that when the sentence contains a small number of triplets, the performances of the two models are not much different, but when the number of triplets increases, the results of our model are much better than the baseline model. Therefore, our model is better at extracting complex triplets than other models.

## 5. Conclusions

In this paper, we propose a relational triplet joint tagging network (RTJTN) which contains a joint tagging of entities and relations and a relational triplet judgment network. Instead of extracting entities and relations in unstructured sentences separately, our tagging method completely combines entity extraction and relation extraction into one task and effectively solves the problems of SPO and EPO. As a consequence, our model can effectively avoid error iteration and get better performance than the baseline model. In addition, we use the RO problem as an indicator of the evaluation model for the first time and our relational triplet judgment layer has achieved excellent results on the RO problem. In the evaluation of NYT, DuIE 2.0, and CMED datasets, our model has also made significant improvements in performance. The results of our model are also better than those of the baseline model in experiments with various overlapping problems and the different number of triplets problems.

## Figures and Tables

**Table 1:** Statistic of the datasets.

Dataset	Train.	Valid.	Test.	Overlapping pattern			Number of triplets				
				SEO	EPO	RO	*N* = 1	*N* = 2	*N* = 3	*N* = 4	*N* ≥ 5
NYT	56195	5000	5000	1297	978	690	3244	1045	312	291	108
DuIE 2.0	173108	20674	50583	8009	2652	3734	12242	4488	1603	1019	1322
CMED	14339	3585	4482	2161	67	1805	1380	779	433	312	681

**Table 2:** Results on the NYT dataset.

Models	Prec	Rec	F1
NovelTagging	62.4	31.7	42.0
CopyRE	61.0	56.6	58.7
GraphRel	63.9	60.0	61.9
CopyMTL	75.7	68.7	72.0
ETL-Span	84.9	72.3	78.1
WDec	**94.5**	76.2	84.4
RSAN	85.7	83.6	84.6
CasRel	89.7	**89.5**	89.6
RTJTN	92.5	89.0	**90.7**

**Table 3:** Results on the DuIE 2.0 and CMED datasets.

Models	DuIE 2.0			CMED		
	Prec	Rec	F1	Prec	Rec	F1
CasRel	70.2	70.0	70.1	56.8	46.7	51.3
RTJTN	**76.6**	**75.6**	**76.1**	**61.8**	**51.8**	**56.4**

**Table 4:** Ablation study on NYT dataset.

Models	Prec	Rec	F1
RTJTN	92.5	89.0	90.7
Relation Positional Attention	91.7	89.0	90.5
Information of Sentence	91.4	89.1	90.2
Maxpooling and Average Pooling	91.2	88.5	89.8
Triplet Judgment Network	87.1	89.5	88.3

**Table 5:** F1 score of sentences with different number of triplets.

Models	Number of triplets														
	NYT					DuIE 2.0					CMED				
	*N* = 1	*N* = 2	*N* = 3	*N* = 4	*N* ≥ 5	*N* = 1	*N* = 2	*N* = 3	*N* = 4	*N* ≥ 5	*N* = 1	*N* = 2	*N* = 3	*N* = 4	*N* ≥ 5
CasRel	**88.2**	90.3	**91.9**	94.2	83.7	68.5	66.9	68.3	70.2	72.1	**46.0**	45.2	47.9	49.5	54.6
RTJTN	**88.2**	**90.6**	91.2	**95.1**	**90.3**	**76.1**	**74.1**	**74.6**	**77.0**	**78.2**	44.4	**49.3**	**56.3**	**58.4**	**64.6**

## Data Availability

The data used to support the findings of this study are included within the article.

## References

[1] Nadeau D., Sekine S. (2007). A survey of named entity recognition and classification. *Lingvisticæ Investigationes. International Journal of Linguistics and Language Resources*.

[2] Zelenko D., Aone C., Richardella A. (2003). Kernel methods for relation extraction. *Journal of Machine Learning Research*.

[3] Yu X., Lam W. Jointly identifying entities and extracting relations in encyclopedia text via a graphical model approach.

[4] Li Q., Ji H. Incremental joint extraction of entity mentions and relations.

[5] Ren X., Wu Z., He W. Cotype: joint extraction of typed entities and relations with knowledge bases.

[6] Gupta P., Schütze H., Andrassy B. Table filling multi-task recurrent neural network for joint entity and relation extraction.

[7] Katiyar A., Cardie C. Going out on a limb: joint extraction of entity mentions and relations without dependency trees.

[8] Wei Z., Su J., Wang Y., Tian Y., Chang Y. (2019). A novel cascade binary tagging framework for relational triple extraction. https://arxiv.org/abs/1909.03227.

[9] Wu S., He Y. Enriching pre-trained language model with entity information for relation classification.

[10] Zhang Y., Yang J. (2018). Chinese NER using lattice LSTM. https://arxiv.org/abs/1805.02023.

[11] Zeng X., Zeng D., He S., Liu K., Zhao J. Extracting relational facts by an end-to-end neural model with copy mechanism.

[12] Nayak T., Ng H. T. Effective modeling of encoder-decoder architecture for joint entity and relation extraction.

[13] Yuan Y., Zhou X., Pan S., Zhu Q., Song Z., Guo L. A relation-specific attention network for joint entity and relation extraction.

[14] Riedel S., Yao L., McCallum A. Modeling relations and their mentions without labeled text.

[15] Miwa M., Sasaki Y. Modeling joint entity and relation extraction with table representation.

[16] Zheng S., Wang F., Bao H., Hao Y., Zhou P., Xu B. (2017). Joint extraction of entities and relations based on a novel tagging scheme. https://arxiv.org/abs/1706.05075.

[17] Fu T.-J., Li P.-H., Ma W.-Y. GraphRel: modeling text as relational graphs for joint entity and relation extraction.

[18] Bekoulis G., Deleu J., Demeester T., Develder C. (2018). Joint entity recognition and relation extraction as a multi-head selection problem. *Expert Systems with Applications*.

[19] Yu B., Zhang Z., Shu X. (2019). Joint extraction of entities and relations based on a novel decomposition strategy. https://arxiv.org/abs/1909.04273.

[20] Liu J., Chen S., Wang B., Zhang J., Li N., Xu T. Attention as relation: learning supervised multi-head self-attention for relation extraction.

[21] Devlin J., Chang M.-W., Lee K., Toutanova K. (2018). Bert: pre-training of deep bidirectional transformers for language understanding. https://arxiv.org/abs/1810.04805.

[22] Radford A., Narasimhan K., Salimans T., Sutskever I. (2018). Improving language understanding by generative pre-training. https://s3-us-west-2.amazonaws.com/openai-assets/research-covers/language-unsupervised/languageunderstandingpaper.

[23] Vaswani A., Shazeer N., Parmar N. (2017). Attention is all you need. https://arxiv.org/abs/1706.03762.

[24] Shaw P., Uszkoreit J., Vaswani A. (2018). Self-attention with relative position representations. https://arxiv.org/abs/1803.02155.

[25] Loshchilov I., Hutter F. (2017). Decoupled weight decay regularization. https://arxiv.org/abs/1711.05101.

[26] Zeng D., Zhang H., Liu Q. Copymtl: copy mechanism for joint extraction of entities and relations with multi-task learning.

